# The Impact of Load-Dump Stress on p-GaN HEMTs Under Floating Gate Condition

**DOI:** 10.3390/mi16121369

**Published:** 2025-11-30

**Authors:** Zhipeng Shen, Yijun Shi, Lijuan Wu, Liang He, Xinghuan Chen, Yuan Chen, Dongsheng Zhao, Jiahong He, Gengbin Zhu, Huangtao Zeng, Guoguang Lu

**Affiliations:** 1 School of Physics and Electronic Science, Changsha University of Science and Technology, Changsha 410114, China; 2 China Electronic Product Reliability and Environmental Testing Research Institute, Guangzhou 511370, China

**Keywords:** load-dump stress, transient charge, floating gate, p-GaN HEMT, capacitive coupling current

## Abstract

This work investigates the impact of load-dump stress on p-GaN HEMTs under floating gate condition. The experiments show that preconditioning the device with a small load-dump stress (150 V, @*t*_d_ = 100 ms and *t*_r_ = 8 ms) enhances its robustness against a larger stress (190 V, @*t*_d_ = 100 ms and *t*_r_ = 8 ms). If a large load-dump stress (≥160 V, @*t*_d_ = 100 ms and *t*_r_ = 8 ms) is applied directly to the device’s drain, the device will burn out. This occurs because the rapidly changing drain voltage during a load-dump event can generate a capacitive coupling current, leading to transient positive charge accumulation in the gate region. Consequently, the channel under the gate is turned on, allowing a large current to flow through it. The coexistence of high current and high voltage leads to substantial Joule heating within the device, resulting in eventual burnout. When a small load-dump stress is initially applied, the resulting charging of electron traps in the gate region increases the threshold voltage. As a result, the device can withstand a larger load-dump stress before the channel turns on, which explains the device’s enhanced robustness. This work clarifies the failure threshold of p-GaN HEMTs under the load-dump stress, providing key support for improving the devices’ reliability in the practical applications. It can provide a basis for adding necessary protective measures in device circuit design, and clarify the triggering voltage threshold of protective measures to ensure that they can effectively avoid device damage due to the load-dump stress.

## 1. Introduction

GaN-based power HEMTs are increasingly adopted in power electronic systems because of their high switching frequency, high power density, and high conversion efficiency [1,2,3,4,5,6,7,8]. Currently, GaN HEMTs are increasingly critical in automotive systems, such as power factor correction (PFC) circuits for onboard chargers (OBCs), DC/DC converters, DC/AC inverters, and LIDAR sensors. Compared to traditional silicon devices, GaN HEMTs in automotive systems offer higher efficiency, smaller size and weight, greater power density, and lower energy loss and electromagnetic interference [9].

However, for p-GaN HEMTs to achieve widespread adoption in automotive electronics, their reliability under various stress conditions must be comprehensively studied. These include electrostatic discharge (ESD), unclamped inductive switching (UIS), surge current, short-circuit, and steady-state stress. Yijun Shi et al. [10] compared the forward/reverse G-to-S ESD robustness of Ohmic-gate and Schottky-gate p-GaN HEMTs, concluding that the Ohmic-gate structure is markedly more robust. Sheng Li [11] investigated the impact of repeated unclamped inductive switching (UIS) stress on p-GaN HEMTs and confirmed that charge trapping near the gate and in the gate-to-drain access region is the primary degradation mechanism. Yinxiang Liu [12] compared the surge current capability of Schottky-gate and Ohmic-gate p-GaN HEMTs in the third quadrant, revealing that the Ohmic-gate structure enables higher surge current by enhancing hole injection from the p-GaN layer into the AlGaN barrier, which promotes electron accumulation in the 2DEG channel. Ning Yang [13] studied the short-circuit robustness of Schottky-gate p-GaN HEMTs under repeated tests and found that charge trapping/detrapping in the p-GaN gate is the primary cause of recoverable degradation under light SC stress. These studies have investigated the reliability and degradation mechanisms of GaN power devices under various stress conditions, providing valuable insights for enhancing their performance in automotive applications. Nevertheless, power devices in automotive electronics face additional challenges, such as load-dump events.

**Figure 1:**
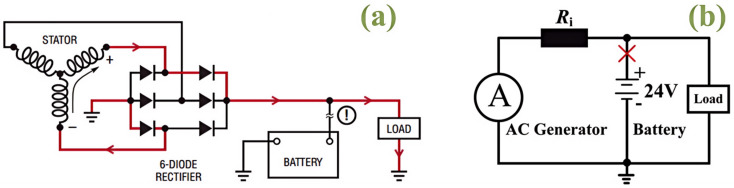
(**a**) Actual circuit diagram of load-dump event. (**b**) Simplified diagram of load-dump event.

As illustrated in Figure 1a, a load-dump event is caused by the abrupt interruption of the charging link between the generator and battery. The consequent release of stored energy creates a transient overvoltage spike, jeopardizing downstream components. This mechanism is depicted in the simplified circuit of Figure 1b. Such overvoltage events primarily lead to insulation breakdown, device failure, and performance degradation in automotive electronic systems [14,15]. Currently, no study has specifically examined the impact of load-dump stress on GaN devices. In practical applications, the gate drive circuit of a p-GaN HEMT may be powered off or damaged, leaving the gate in a floating state. Our team will conduct in-depth research on the impact of load-dump stress on p-GaN HEMTs under floating gate condition. Accordingly, further research is essential to enhance the reliability of GaN devices in automotive electronics.

This work investigates the reliability of commercial 100 V/16 A p-GaN HEMTs under load-dump stress. The paper is structured as follows: Section 2 describes the experimental setup, Section 3 analyzes and discusses the results, and Section 4 concludes the study.

## 2. Experimental Setup

**Figure 2:**
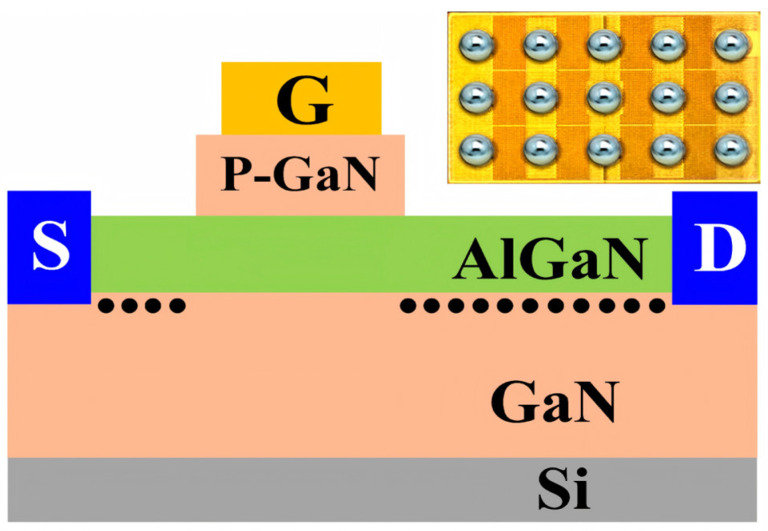
Cross-sectional diagram of the EPC2045 structure. The inset in Figure is the physical object image of EPC2045.

**Figure 3:**
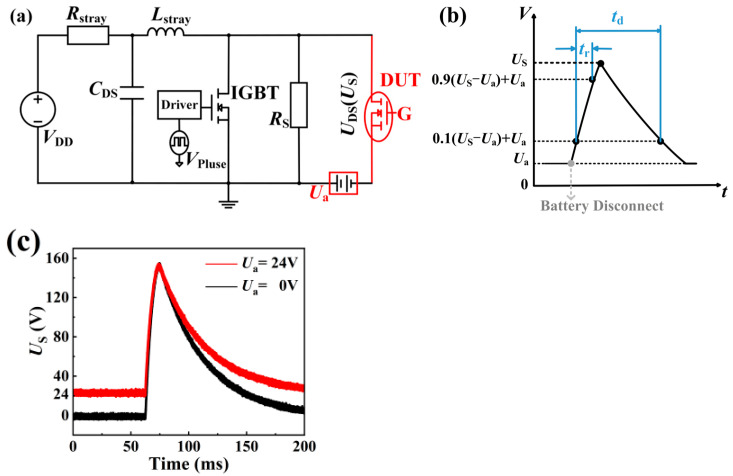
(a) Test circuit topology diagram. The principle of stress testing is that when the IGBT is turned on (simulating battery connection), energy is stored in the load inductor. During the IGBT turn-off process (simulating battery disconnection), the energy stored in the load inductor is released, generating a transient overvoltage. (**b**) Simulated waveform diagram of load-dump stress. (**c**) Actual waveform of the load-dump stress generated by the LDS 200N.

Figure 2 illustrates the cross-section of the device under test (EPC2045), which has a rated drain current of 16 A and a voltage rating of 100 V. The load-dump stress is generated by the load-dump test equipment (LDS 200N, 3CTEST Suzhou, China). Figure 3a shows the circuit topology of the load-dump test equipment, which consists of a charging circuit and a discharging circuit. When the IGBT is turned on (simulating battery connected), energy is stored in the load inductor. During the IGBT turn-off process (simulating battery disconnection), the energy stored in the load inductor is released, generating a transient overvoltage. The resulting load-dump stress waveforms are presented in Figure 3b,c. The voltage *U*_a_ simulates the vehicle’s operating state prior to a load-dump event. A value of *U*_a_ = 0 V represents an event in a stationary car, while *U*_a_ = 24 V corresponds to a high-power automotive supply system, and simulates a sudden load-dump event in a running car. Consequently, the device under test experiences a load-dump stress superimposed on the bias voltage *U*_a_. In addition to applying repeated stress, the LDS 200 N system can also precisely control the output waveform’s pulse width (*t*_d_) and rise time (*t*_r_). This work employs a 24 V system with a 5 A pulse mode, a td of 100 ms, and a tr of 8 ms. This work comprises two experimental categories: (1) a degradation test, where the load-dump stress is applied in a stepwise manner from 150 V to 190 V; and (2) a failure test, where a single, high-magnitude stress of 160 V or 190 V is directly applied. The experimental type is shown in Table 1. After each application of load-dump stress, the Agilent B1505 records device characteristics, including threshold voltage (*V*_TH_) and drain current (*I*_DS_). Recovery characteristics are also measured to assess the resilience of the devices under floating gate conditions and to investigate the underlying degradation mechanisms.

## 3. Results and Discussion

### 3.1. Impact of Load-Dump Stress on p-GaN HEMT Under Floating Gate Conditions at U_a_ = 0 V

**Figure 4:**
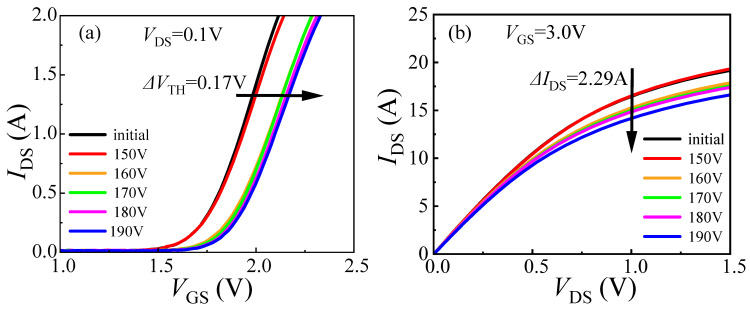
Degradation of DUT-1 (*U*_a_ = 0 V) characteristics after applying stress from 150 V to 190 V. (**a**) Transfer characteristic; (**b**) output characteristic.

**Figure 5:**
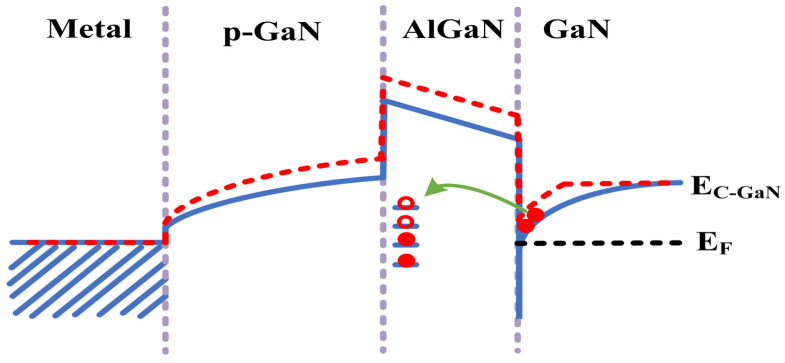
The energy band diagram shows that the electron traps in the gate region of the device are activated when a small load-dump stress is applied initially. These traps capture electrons, resulting in a localized charging effect in the gate region.

**Figure 6:**
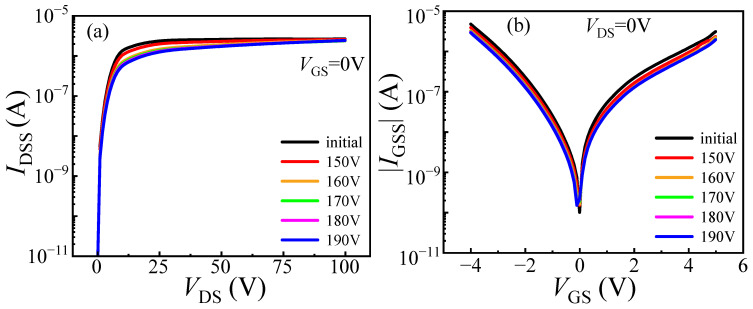
Degradation of DUT-1 (*U*_a_ = 0 V) characteristics after applying stress from 150 V to 190 V. (**a**) Drain leakage current; (**b**) gate leakage current.

The degradation of DUT-1 was characterized under progressively increasing load-dump stress from 150 V to 190 V in 10 V steps. The gate voltage at *I*_DS_ = 100 mA and *V*_DS_ = 0.1 V is defined as *V*_TH_. The on-state current at *V*_DS_ = 1 V and *V*_GS_ = 3 V is defined as *I*_DS_. When the applied stress increases to 190 V, a significant rise in *V*_TH_ is observed, from its initial value of 1.64 V to 1.81 V, corresponding to a change of 0.17 V, as illustrated in Figure 4a. This increase is attributed to the trapping of electrons in the gate and its adjacent region caused by load-dump stress. This process raises the energy band of the channel, requiring a higher gate bias voltage to open the channel (see Figure 5). Meanwhile, the value of *I*_DS_ decreases from 16.76 A to 14.47 A, corresponding to a change of 2.29 A. This occurs because the traps with electrons trapped can repel the electrons in the channel, resulting in a reduction in the two-dimensional electron gas (2DEG) density in the channel, which in return reduces the on-state current of the device [16], indicating that the device requires a higher gate voltage to achieve the same on-state current after the application of load-dump stress. In addition to the changes in *V*_TH_ and *I*_DS_, the leakage currents, including gate leakage current (*I*_GSS_) and drain leakage current (*I*_DSS_), are also measured after the application of load-dump stress. As shown in Figure 6, with the increase in applied stress, both *I*_GSS_ and *I*_DSS_ decrease slightly. This result confirms the earlier hypothesis that electron trapping increases the effective barrier thickness in and around the gate region, leading to the observed reduction in *I*_GSS_ and *I*_DSS_.

### 3.2. Impact of Load-Dump Stress on the p-GaN HEMT Under Floating Gate Conditions at U_a_ = 24 V

**Figure 7:**
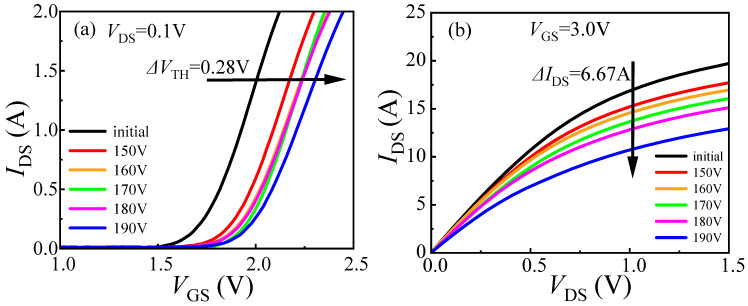
Degradation of DUT-2 (*U*_a_ = 24 V) characteristics after applying stress from 150 V to 190 V. (**a**) Transfer characteristic; (**b**) output characteristic.

**Figure 8:**
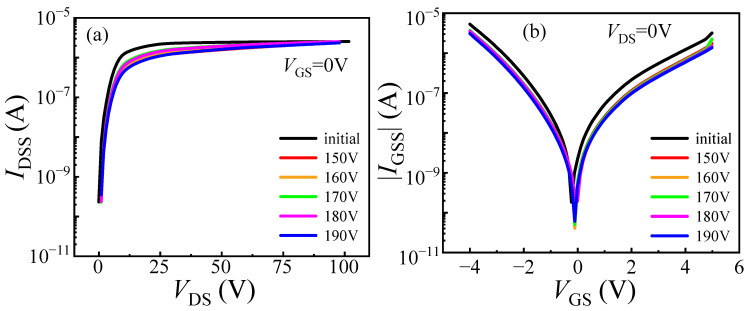
Degradation of DUT-2 (*U*_a_ = 24 V) characteristics after applying stress from 150 V to 190 V. (**a**) Drain leakage current; (**b**) gate leakage current.

**Figure 9:**
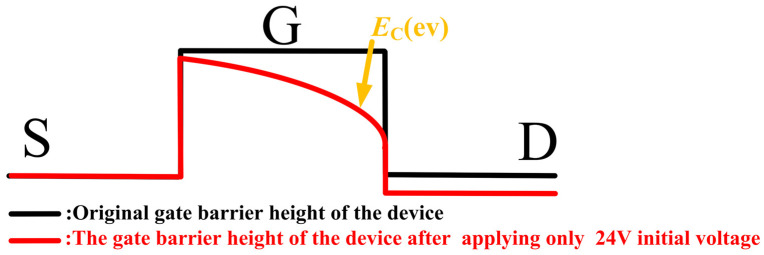
The initial voltage of 24 V reduces the height of the gate barrier layer.

**Figure 10:**
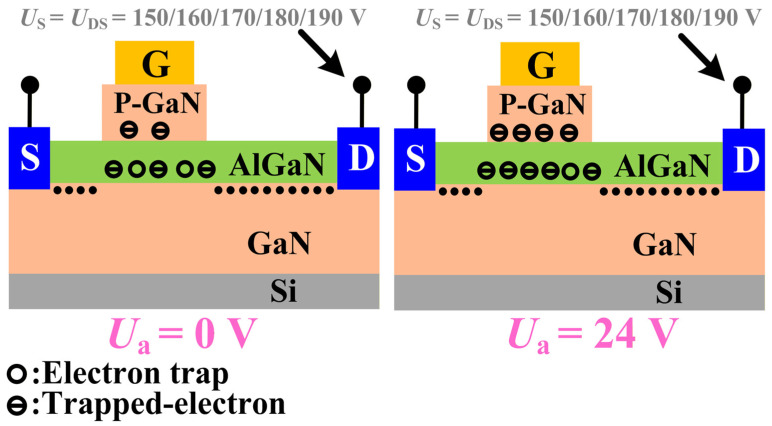
The relationship between the initial voltage (0 V/24 V) and the number of trapped electrons.

DUT-2 was tested following the same experimental steps as DUT-1, but with an initial voltage of 24 V. As shown in Figure 7, this resulted in a *V*_TH_ shift of 0.28 V and an *I*_DS_ decrease of 2.29 A. As shown in Figure 8, both *I*_GSS_ and *I*_DSS_ for DUT-2 display only marginal reductions, a trend consistent with the behavior observed in DUT-1. However, the changes in *V*_TH_ and *I*_DS_ are more pronounced in DUT-2 than in DUT-1. This is attributed to the application of the initial voltage of 24 V to the drain electrode, which establishes a baseline electric field between the drain and gate electrodes. The 24 V initial voltage creates a relatively high electric field at the gate edge, causing the carriers in this region to reach higher energy states. When load-dump stress is applied, the channel carriers can easily overcome the energy barrier and then be captured by the traps in the barrier layer. Simultaneously, the initial voltage of 24 V reduces the barrier height in the gate region to a certain extent [17,18], as shown in Figure 9. Consequently, the superposition of load-dump stress on the 24 V bias enables a greater number of channel carriers to surmount the barrier layer and become trapped. Consequently, the device exhibits a more pronounced threshold voltage shift. This correlation between the initial voltage (0 V/24 V) and the extent of electron trapping is clearly demonstrated in Figure 10.

**Figure 11:**
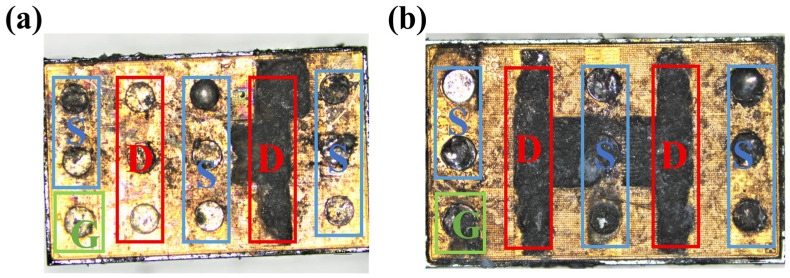
Observe the solder pad at the bottom of the device under the optical microscope. (**a**) DUT-3 (*U*_S_ = 160 V); (**b**) DUT-4 (*U*_S_ = 190 V). (green G: Gate, blue S: Source, red D: Drain).

**Figure 12:**
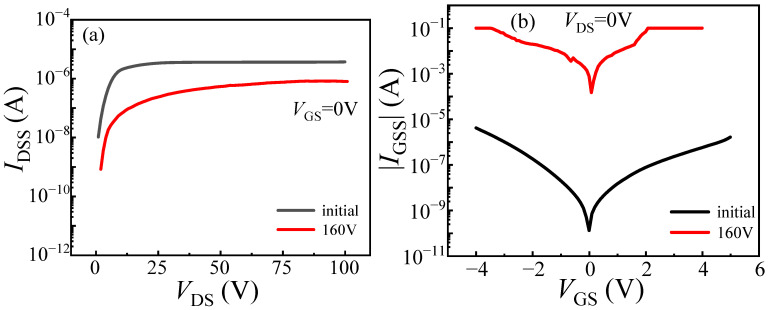
With load-dump stress of 160 V applied directly to DUT-3 drain electrodes. (**a**) Drain leakage current; (**b**) gate leakage current.

**Figure 13:**
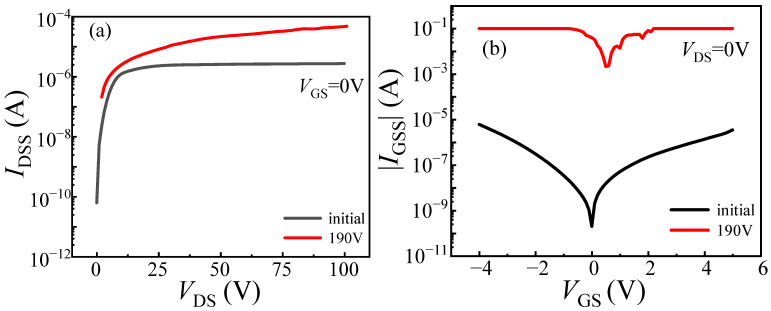
With load-dump stress of 190 V applied directly to DUT-4 drain electrodes. (**a**) Drain leakage current; (**b**) gate leakage current.

**Figure 14:**
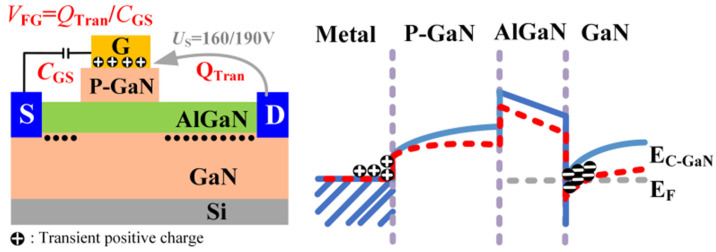
The capacitive coupling current induces transient positive charge in the floating gate p-GaN structure, and the accumulated positive charge lowers its energy band.

**Figure 15:**
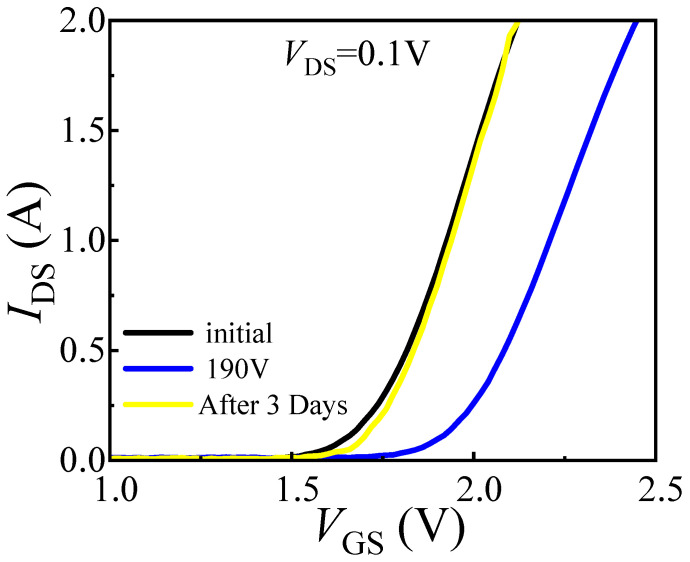
After DUT-2 was subjected to 150 to 190 V load-dump stress, its transfer characteristics were restored within three days.

The failure characteristics under single load-dump stress were further investigated by directly applying 160 V to DUT-3 and 190 V to DUT-4. DUT-4 failed immediately after a direct 190 V load-dump stress was applied to its drain. Similarly, DUT-3 also suffered immediate failure upon the application of a 160 V stress. To analyze the burnout mechanisms, optical microscope is employed to inspect the bottom pads of the devices. As shown in Figure 11a, DUT-3 exhibits severe damage to the drain pad, while the gate pad remains undamaged. In contrast, DUT-4 (Figure 11b) suffered more extensive damage, with complete destruction of the drain pad and partial damage to the gate pad. As shown in Figure 12b, a 160 V load-dump stress causes a sharp increase in *I*_GSS_ for DUT-3. For DUT-4, both *I*_GSS_ and *I*_DSS_ significantly increase after applying 190 V load-dump stress (Figure 13). These results demonstrate that direct exposure to high magnitude load-dump stress leads to severe degradation in both device performance and structural integrity. This failure is initiated by a capacitive coupling current from the drain to the gate and then to the source, induced by the rapid voltage transient on the drain [19,20]. This current leads to the accumulation of transient positive charge at the gate, which lowers the channel energy band (Figure 14). Once the load-dump stress exceeds the critical level, the channel beneath the gate will open. At this point, both high current and high voltage will exist in the gate region, resulting in a large amount of Joule heat and irreversible damage. In contrast, DUT-2 survives the stepwise increase in load-dump stress from 150 V to 190 V without failure and recovers its electrical properties within approximately three days (Figure 15). This is because after initially applying a small load-dump stress, the electron traps in the gate region capture electrons, leading to localized charge effects in the gate region. The capture of electrons by traps in the gate region increases the local charge density. This, in turn, causes an upward shift in the channel energy band, thereby raising the gate bias required for channel formation, resulting in an increase in *V*_TH_. Consequently, the device can withstand a larger load-dump stress before the channel turns on.

## 4. Conclusions

This work investigates the impact of load-dump stress on floating gate p-GaN HEMT, focusing on analyzing the degradation and recovery processes of the device, and investigating potential physical mechanisms. The results demonstrate that the device undergoes burnout when directly subjected to larger load-dump stress (160 V, @*t*_d_ = 100 ms and *t*_r_ = 8 ms). This is because the rapidly changing voltage at the drain electrode generates a capacitive coupling current that flows from the drain to the gate and subsequently to the source electrode, resulting in the accumulation of transient positive charge in the gate region. As a result, the energy band in the channel below the gate is lowered, causing the channel to open and allowing a large current to pass through. The combined presence of large current and high voltage generates substantial Joule heat within the device, ultimately causing burnout. However, if a relatively small load-dump stress (150 V, @*t*_d_ = 100 ms and *t*_r_ = 8 ms) is initially applied, the device will be able to withstand a larger load-dump stress (190 V, @*t*_d_ = 100 ms and *t*_r_ = 8 ms). This is due to the initial application of a smaller load-dump stress, which causes the electron traps in the gate region to accumulate charge, resulting in an increase in threshold voltage. As a result, a larger load-dump stress is required to turn on the device channel, enabling the device to withstand greater stress. Additional protective circuits are needed to be added to prevent damage to the components. Considering that when the device is damaged due to load stress, the stress value usually exceeds the device’s rated voltage. Therefore, an overvoltage protection circuit with a trigger voltage equal to or slightly lower than the device’s rated voltage should be used. For instance, a TVS clamping diode (VBR = 150 V) can be connected in parallel across the drain-source terminals of the device to provide protection.

## Figures and Tables

**Table 1:** Experiment type.

Type	Degradation Experiment	Failure Experiment
Application method	Step by step pressurization	Single pressurization
*U*_S_	150/160/170/180/190 V	160 V or 190 V

## Data Availability

The original contributions presented in this study are included in the article. Further inquiries can be directed to the corresponding authors.

## References

[1] Miyamoto H., Okamoto Y., Kawaguchi H., Miura Y., Nakamura M., Nakayama T., Masumoto I., Miyake S., Hirai T., Fujita M. Atsushi Enhancement-mode GaN-on-Si MOS-FET using Au-free Si process and its operation in PFC system with high-efficiency. Proceedings of the 2015 IEEE 27th International Symposium on Power Semiconductor Devices & IC’s (ISPSD).

[2] Amirahmadi A., Domb M., Persson E. High power density high efficiency wide input voltage range LLC resonant converter utilizing E-mode GaN switches. Proceedings of the 2017 IEEE Applied Power Electronics Conference and Exposition (APEC).

[3] Chen T., Yu R., Huang Q., Huang A.Q. A single-stage bidirectional dual-active-bridge AC-DC converter based on enhancement mode GaN power transistor. Proceedings of the 2018 IEEE Applied Power Electronics Conference and Exposition (APEC).

[4] Qian W., Lu J., Bai H., Averitt S. (2019). Hard-Switching 650-V GaN HEMTs in an 800-V DC-Grid System with No-Diode-Clamping Active-Balancing Three-Level Topology. IEEE J. Emerg. Sel. Top. Power Electron..

[5] Yang S., Han S., Sheng K., Chen K.J. (2019). Dynamic On-Resistance in GaN Power Devices: Mechanisms, Characterizations, and Modeling. IEEE J. Emerg. Sel. Top. Power Electron..

[6] Moradisizkoohi H., Elsayad N., Shojaie M., Mohammed O.A. (2019). PWM Plus Phase-Shift-Modulated Three-Port Three-Level Soft-Switching Converter Using GaN Switches for Photovoltaic Applications. IEEE J. Emerg. Sel. Top. Power Electron..

[7] Martínez P.J., Miaja P.F., Maset E., Rodríguez J. (2019). A Test Circuit for GaN HEMTs Dynamic *R*_ON_ Characterization in Power Electronics Applications. IEEE J. Emerg. Sel. Top. Power Electron..

[8] Huang Q., Huang A.Q. (2020). Variable Frequency Average Current Mode Control for ZVS Symmetrical Dual-Buck H-Bridge All-GaN Inverter. IEEE J. Emerg. Sel. Top. Power Electron..

[9] Favero D., Marcuzzi A., De Santi C., Meneghesso G., Zanoni E., Meneghini M. GaN-on-Si Power HEMTs for Automotive: Current Status and Perspectives. Proceedings of the 2023 AEIT International Conference on Electrical and Electronic Technologies for Automotive (AEIT AUTOMOTIVE).

[10] Shi Y., He Z., Huang Y., Cai Z., Chen Y., Cheng L., Chen W., Sun R., Liu C., Lu G. (2023). A Comparative Study on G-to-S ESD Robustness of the Ohmic-Gate and Schottky-Gate p-GaN HEMTs. IEEE Trans. Electron Devices.

[11] Li S., Liu S., Zhang C., Li N., Tao X., Wei J., Zhang L., Sun W. (2021). Investigations on Electrical Parameters Degradations of p-GaN HEMTs Under Repetitive UIS Stresses. IEEE J. Emerg. Sel. Top. Power Electron..

[12] Liu Y., Yang S., Han S., Sheng K. (2019). Investigation of Surge Current Capability of GaN E-HEMTs in The Third Quadrant: The Impact of P-GaN Contact. IEEE J. Emerg. Sel. Top. Power Electron..

[13] Yang N., Pan C., Wu Z., Bai P., Chen K., Zhu L., Zhou C., Zhang B., Zhou Q. (2024). Study of the Short-Circuit Capability and Device Instability of p-GaN Gate HEMTs by Repetitive Short-Circuit Stress. IEEE Trans. Power Electron..

[14] Texas Instruments (2013). Load Dump and Cranking Protection for Automotive Backlight LED Power Supply.

[15] Efland T., Manternach M., Marshall A., Mings J. The load dump (automobiles). Proceedings of the IEEE Workshop on Electronic Applications in Transportation.

[16] Raja P.V., Dupouy E., Bouslama M., Sommet R., Nallatamby J.-C. (2022). Estimation of Trapping Induced Dynamic Reduction in 2DEG Density of GaN-Based HEMTs by Gate-Lag DCT Technique. IEEE Trans. Electron Devices.

[17] Nuo M., Wei J., Wang M., Yang J., Wu Y., Hao Y., Shen B. (2022). Gate/Drain Coupled Barrier Lowering Effect and Negative Threshold Voltage Shift in Schottky-Type p-GaN Gate HEMT. IEEE Trans. Electron Devices.

[18] Nuo M., Wu Y., Yang J., Hao Y., Wang M., Wei J. (2023). Time-Resolved Extraction of Negatively Shifted Threshold Voltage in Schottky-Type p-GaN Gate HEMT Biased at High VDS. IEEE Trans. Electron Devices.

[19] Shi Y., Chen Y., Huang Y., He Z., Chen W., Sun R., Yao B., Wang H., Xiao Q., Lu G. (2022). A Novel Gate-to-Source ESD Protection Clamp for GaN HEMT. IEEE Trans. Electron Devices.

[20] Wen Q., Zhou L., Meng X., Feng S., Zhang Y. (2024). Trap Location and Stress Degradation Analysis of GaN High Electron Mobility Transistors Based on the Transient Current Method. IEEE Trans. Device Mater. Reliab..

